# D-Dimer, Fibrinogen, and IL-6 in COVID-19 Patients with Suspected Venous Thromboembolism: A Narrative Review

**DOI:** 10.2147/VHRM.S280962

**Published:** 2020-11-13

**Authors:** Islam Eljilany, Abdel-Naser Elzouki

**Affiliations:** 1 Qatar University, College of Pharmacy, Doha, Qatar; 2 Hamad Medical Corporation, Hamad General Hospital, Department of Medicine, Doha, Qatar; 3 Department of Medicine, Qatar University, College of Medicine, Doha, Qatar

**Keywords:** COVID-19, SARS-CoV-2, D-dimer, fibrinogen, IL-6, venous thromboembolism

## Abstract

Coronavirus disease 2019 (COVID-19) emerged from the West District of Southern China Seafood Wholesale Market in late December 2019 and has been declared a global pandemic by the World Health Organization (WHO). Infection with severe acute respiratory syndrome coronavirus (SARS-CoV-2) presents with upper respiratory symptoms like cough, fever, and lethargy. At the same time, in later stages, critical COVID-19 patients develop acute respiratory distress syndrome (ARDS), venous thromboembolism (VTE), and multiple organ failure from cytokine storm and coagulation hyperactivity. Primary manifestations of thrombotic events include deep vein thrombosis (DVT), disseminated intravascular coagulation (DIC) and pulmonary embolism (PE). Initial coagulopathy in COVID-19 patients presents with elevated fibrin degradation products, especially D-dimers. In contrast, late presentations show evidence of prolonged prothrombin time (PT) and activated partial thromboplastin (aPTT), increased platelets, and fibrinogen levels. Diagnosis and monitoring of disease progression are done by regular screening of laboratory parameters, including D-dimer and fibrinogen. Management of coagulopathy in COVID-19 patients is like that of critically ill patients, including thromboprophylaxis. Coagulopathy is a poor prognostic factor, and optimum strategies should be developed for early diagnosis, prevention, and prompt treatment of VTE in COVID-19 patients. Thrombosis prophylaxis with low molecular weight heparin (LMWH) has shown beneficial results in preventing coagulopathy a reducing risk of mortality due to thrombotic events. We will discuss VTE in COVID-19 patients highlighting the role of D-dimer, fibrinogen, and interleukin-6 (IL-6).

## Introduction

World Health Organization (WHO) identified coronavirus disease 2019 (COVID-19) as a new pandemic during an investigation into an outbreak in December 2019 in the seafood market of Wuhan, China. The outbreak spread to 187 countries worldwide within only 3 months with high morbidity and mortality.^1^ Since the outbreak, the pandemic has killed more than 757,471 patients worldwide until August 14th, 2020.^2^ COVID-19 is acute interstitial pneumonia resulting from infection of severe acute respiratory syndrome coronavirus 2 (SARS-CoV-2), a virus with a single-stranded RNA genome and characteristic surface spike proteins. Same as other SARS-like viruses, bats are said to be the source of the SARS-CoV-2 virus.^3^

Although COVID-19 primarily causes lower respiratory tract infection presenting as cough, fever, dyspnea, and lethargy, it can also have cardiovascular and immune system complications like single or multi-organ failure and disseminated intravascular coagulation (DIC).^4^

The current review summarizes the risk of venous thromboembolism (VTE) in COVID-19 patients, its prevalence, and incidence. Besides, the diagnosis and pathogenesis of VTE in such patients are reviewed. Also, the manuscript focuses on the role of D-dimer, fibrinogen, and interleukin-6 (IL-6) in development and investigating VTE in COVID-19 patients. Finally, the management of these cases is addressed.

## Methodological Consideration

A comprehensive assessment of the published evidence on (MEDLINE with PubMed interface, date of the last search: July 31, 2020) was provided to accommodate the inclusion of relevant articles for this review.

## Risk of VTE in COVID-19 Patients

VTE is one of the severe complications identified in critical COVID-19 patients and coagulopathy resulting in VTE and DIC has been reported as the primary cause of death in critical patients.^5^ Numerous hemostatic cellular and plasmatic elements interact to trigger inflammatory and immune cascade leading to VTE in the presence of sepsis and acute respiratory distress syndrome (ARDS).^6^ Till 17th April 2020, 41% of COVID-19 patients had complications like ARDS and VTE, surprising the homeostatic and intensive care community regarding their high incidence in these patients.^7^

Although most of the patients completely recover from the infection, older patients with associated comorbidities like myocardial infarction (MI), diabetes mellitus, hypertension, stroke, and immunosuppression, have a poor prognosis and high risk of complications. Various risk factors associated with VTE in COVID-19 patients have been shown in Table 1. These risk factors are summarized under three categories: firstly, patient-related characteristics. Secondly, hospital-related factors. Lastly, SARS-COV2 specific elements. All these factors can lead to a heterogenicity in reporting VTE phenotype (isolated or concurrent deep vein thrombosis (DVT) and pulmonary embolism (PE)).^8^ Management of such complicated patients is challenging and requires high medical precision and timely nebulization with high-flow oxygen, inhalations, dexamethasone, vasopressors, and even mechanical ventilation.^9^,^10^

**Table 1:** Risk Factors for Venous Thromboembolism (VTE)

COVID-19-Related Risk Factors	Variables
Age	≥70 year
Gender	Males > females
Obesity	BMI > 30
Cancer	Active or not
Comorbidities	Hypertension, CVD, diabetes, stroke, CKD
Medical ICU admission	18.5%
Inflammation	Existing or not
Cytokine release syndrome (cytokine storm)	High-grade fevers, hypotension, multi-organ dysfunction
Lung injury	Pre-existing or not

**Abbreviations:** BMI, body mass index; CVD, cardiovascular disease; CKD, chronic kidney disease; ICU, intensive care unit.

## Prevalence and Incidence of VTE in COVID-19 Patients

VTE complications were first reported in up to 30% of COVID-19 patients admitted to medical intensive care unit (ICU) in China and the Netherlands.^11^,^12^ Subsequent studies in critically ill COVID-19 patients from the USA, Italy and France demonstrated thrombosis in intravenous catheters and arterial vascular occlusive events, including acute MI, acute limb ischemia, and stroke.^13–20^ A latest review found that the prevalence of DVT and PE in COVID-19 patients in ICU fluctuates from 0%- 54%.^8^ On the other hand, a group of Italian researchers at the School of Medicine, University of Cattolica del Sacro Cuore who observed that the incidence of DVT in non-ICU hospitalized for COVID-19 patients, is 11.9%.^21^ These results were matching to a recent meta-analysis that investigated the risk of VTE in COVID-19 patients found that the incidence of VTE is 26%, while DVT and PE were 12% and 14%, respectively.^22^ A study that investigated patients from 2 leading hospitals in Wuhan, China found that out of 184 ICU patients, just more than a courter (27%) developed VTE, while less than 5% of patients developed arterial Thromboembolism (TE).^5^ In the same line, Middeldorp et al23 showed DVT has been formed in the same percentage (27%) of ICU and 1.6% of non-ICU patients with COVID-19. A Dutch study of 184 patients with COVID-19 pneumonia admitted to an ICU found a 49% cumulative incidence of thrombotic complications, mainly changes suggestive of pulmonary embolism (PE) observed on Computed tomography (CT) pulmonary angiogram.^12^ Other studies from the Netherlands and France have also suggested that VTE occurs in 20–30% of critically ill COVID-19 patients, even with prophylaxis.^23^,^24^ Pneumonia, old age, PT >3 s, and aPTT>5 s were individual predictors of VTE in these patients. Khan et al^7^ supported these results by reporting lower extremity VTE in 20 out of 81 COVID-19 patients with mean age 59.9 years, who were not given preventive anticoagulation. Laboratory tests of these patients showed lymphopenia, prolonged aPTT, and high D-dimer values. Therefore, VTE was inevitable in critical COVID-19 patients and developed much more frequently than SARS unless managed timely.

## Hematological Manifestations of COVID-19 Patients

The spectrum of clinical presentation of COVID-19 patients is ranged from mild symptoms to severe respiratory failure with multiple organ failure. A substantial number of COVID-19-infected individuals remain asymptomatic. The definition of asymptomatic is positive COVID-19 test but clinical symptoms and imaging are normal. Patient with mild symptoms has acute upper respiratory tract infection and/or digestive symptoms. While moderate cases are suffering from Pneumonia without obvious hypoxemia or chest CT with lesions. On the other hand, severe patients are facing Pneumonia with hypoxemia. Lastly, critical COVID-19 case usually has ARDS, may have shock, heart failure, encephalopathy, coagulation dysfunction, myocardial injury and acute kidney injury.^25^ The symptoms usually start on the 7th post-infection day, shortness of breath on 8th day, pneumonia on the 9th day, and ARDS requiring ICU admission on the 10th to 11th day.^3^

COVID-19 patients may present with some laboratory abnormalities and VTE complications.^26^ The most frequently deranged laboratory parameters include prolonged prothrombin time (PT) and activated partial thromboplastin time (aPTT). This followed by a reduction in both due to coagulopathy consumption, increased fibrinogen, increased platelet count, three-fold rise in D-dimer levels, and elevated C-Reactive Proteins.^3^ Table 2 summarizes the potentially useful laboratory tests in COVID-19 patients.

**Table 2:** Summary of Potentially Useful Laboratory Tests and Their Expected Outcome in COVID-19

Tests Which Their Level is Expected to Increase	Tests Which Their Level is Expected to Decrease
aPTT (in acute phase) ALT and AST CRP D-dimer Fibrinogen (in acute phase) LDH PT (in acute phase) CBC (platelets and lymphocytes in acute stage)	Albumin aPTT (in late phase) CBC (platelets and lymphocytes in late stage) Fibrinogen (in late phase) PT (in late phase)

**Abbreviations:** aPTT, activated partial thromboplastin; ALT, alanine aminotransferase; AST, aspartate aminotransferase; CBC, complete blood count; CRP, C-reactive protein; LDH, lactate dehydrogenase; PT, prothrombin time.

## Diagnosis of VTE and Its Difficulties in COVID-19 Patients

The incidence and prevalence of VTE are strongly correlated with SARS-CoV-2 infection. Therefore, its diagnosis and treatment are crucial for reducing mortality.^3^ However, it might be challenging to diagnose VTE in COVID-19 because of a prolonged disease course during which management protocols like oxygen supply and intubation can mask the signs and symptoms indicative of VTE. Diagnosis can be missed in otherwise asymptomatic patients.^7^ As a result, the derangement of numerous laboratory parameters is indicative of imminent or life-threatening VTE, and repeat testing of these parameters should be done regularly.^3^

Alterations in various hemostatic biomarkers shown in Table 2 play a role in the thromboembolic stage of the disease. Prolonged PT and aPTT suggest coagulation activation, while fibrinogen and platelet levels are a part of acute phase changes.^7^

In the late thrombolytic stages of the DIC; PT, aPTT, fibrinogen, and platelets decrease due to consumptive coagulopathy. Thrombin-antithrombin complex increases, fibrin-degradation products, especially, D-dimers, are also elevated. The degree of alteration in levels indicates the severity of disease outcome.^27^

## Pathogenesis of COVID-19

In severe infection with sepsis and ARDS, DIC develops due to a complex interplay between cellular and plasmatic elements involved in inflammation and thrombin generation, collectively known as “thrombo-inflammation”.^28^ SARS-CoV-2 consists of four structural proteins; spike (S), membrane (M), envelop (E), and nucleocapsid (N). The specific surface of S proteins has a particular affinity for hosting angiotensin-converting enzyme-2 (ACE-2) receptors.^3^

The life cycle of the virus with the host consists of five steps. Starting with the attachment step in which the virus is transmitted through respiratory droplets, enters the bloodstream and mainly gets lodged into the tissues expressing ACE-2 receptors, including type 2 alveolar cells of lungs, gastrointestinal tract, endothelial cells of heart and blood vessels, pericytes, adipocytes, and neural cells where virus binds to host ACE-2 receptors.^4^ Then, the virus penetrates host cells through membrane fusion or endocytosis. After that, the viral contents are released inside the host cells, and its viral RNA enters the nucleus for replication by using the viral mRNA for the manufacturing of viral proteins; this step is called biosynthesis. Later, new viral particles are made and, finally, it released. Viral adhesion to ACE-2 receptors followed by viral replication causes inflammatory cell infiltration, endothelial cell apoptosis and microvascular thrombosis. Gupta et al^26^ illustrated this process in his recent article titled “Extrapulmonary manifestations of COVID-19”.

## D-Dimer Levels in COVID-VTE Patients

D-dimer is a fibrin-degradation product which is increased in thrombotic events, indicating fibrinolysis.^29^ Scientists studied D-dimer levels of critical COVID-19 pneumonia and their association with a high risk of VTE, disease severity, and risk of mortality.^30^ Raised D-dimer values contributed to poor prognosis and high mortality in such patients.^31^ Such high values of D-Dimer can be attributed to the activation of coagulation cascade Secondary to Systemic Inflammatory Response Syndrome (SIRS) in COVID-19 patients.^32^ Zhou et al^33^ demonstrated the link between high D-dimer levels and disease severity in 129 COVID-19 patients admitted to the Shanghai Public Health Clinical Center. According to the results, the rise in D-dimer levels in mild and severe infection was <2 × Upper Limit of Normal (ULN) and >10 ULN, respectively. This is evident when Mucha et al^34^ defined the threshold value of D-dimer for high-risk patients as six times the upper limit, ie, 3000 ng/mL Fibrinogen Equivalent Units [FEU]. Likewise, Artifoni’s research^35^ showed a positive predictive value of 44% and 67% for D-dimer level ≥1.0 µg/mL and ≥3.0 µg/m, respectively, in his cohort of 65 out of 71 VTE COVID-19 patients. The previous link has been seen in VTE subtypes (DVT and PE). For example, a study by Demelo-Rodríguez et al^29^ also confirmed the link between D-dimer levels and risk of DVT through a retrospective analysis of 156 non-ICU COVID-19 patients. He found that D-dimer levels in these patients with DVT were 4527 ng/mL as compared to 2050 ng/mL in those without DVT. Another cross-sectional study found that admission levels of D-dimer were independently associated with a higher risk for proximal DVT while, D-dimer, urea, respiratory rate, blood pressure, and 65 years of age or older and (CURB-65) score were independently associated with a higher risk of distal DVT in COVID-19 patients.^36^ Results reported the incidence of DVT is 88.5% of patients with D-dimer >1.0 μg/mL as compared to 15.9% COVID-19 patients with d-dimer <1.0 μg/mL.

The association between D-dimer PE has been confirmed by French research,^32^ which demonstrated the link between the rise in D-dimer levels and risk of PE through a retrospective analysis of CT angiogram of the chest of suspected and confirmed COVID-19 patients with PE and compared them with their D-dimer levels. In the findings, D-dimer levels greater than 2660 µg/L showed 100% sensitivity and 67% specificity for PE. High D-dimer levels have low specificity value for VTE because they are increased in many conditions like pregnancy, sepsis, malignancy, and post-operative states. However, D-dimer levels are highly sensitive (80–100%) for VTE. Therefore, normal levels rule out VTE.^37^

All the studies, as mentioned above, show a strong association between D-dimer levels and incidence of all types of VTE in COVID-19 patients. Consequently, call for the daily assessment of D-dimer to assess disease progress in severely infected patients is preferable. Thus, anticoagulation therapy should be started once the D-dimer levels are >1000 ng/mL.^7^

## IL-6 in COVID-19 VTE Patients

Infection by virus, bacteria, or fungi induces host defense mechanisms, which subsequently increases cellular and humoral immune responses by activation of coagulation cascade and thrombin generation.^4^ Mucha’s study^34^ explained cytokine storm as the hallmark of COVID-19 pathophysiology and it is characterized by high levels of inflammatory markers, including IL-1 and IL-6, that promote thrombosis by activating platelets, endothelium, monocytes, and the tissue factor VIIa pathway. Besides, they inhibit fibrinolysis and natural anticoagulants, including protein C and S. Inflammatory chemokines cause localized lung injury by damaging alveoli, causing endothelial apoptosis, dysregulating coagulation, and inducing pulmonary fibrinolysis. Infection by SARS-CoV-2 induces similar complex systemic inflammatory responses releasing proinflammatory cytokines that have numerous pleiotropic effects, including activation of the coagulation cascade which interacts with coagulopathies to form a vicious cycle directly correlated with poor prognosis.^33^ Ranucci et al^28^ presented the link between IL-6 levels in COVID-19 patients and ARDS requiring mechanical ventilation. The results showed a proportional increase between IL-6 and fibrinogen levels, demonstrating the link between inflammation and procoagulant changes. A parallel analysis revealed an elevation in IL-6 associated with increased mortality in COVID-19 patients.^5^ Excessive IL-6 signaling also causes multiple organ damage through T-cell maturation, expression of vascular endothelial growth factor (VEGF), increase in vascular permeability, and decrease in myocardial contractility. Therefore, IL-6 plays a significant role in the pathogenesis of VTE in COVID-19 patients.^27^ Excessive signaling of it promotes coagulation cascade and development of VTE. Consequentially, for patients with elevated IL-6, timely administration of the IL-6 inhibitor tocilizumab may improve cytokine release syndrome and reduce the risk of DIC.^33^

## Fibrinogen Levels in COVID-19 VTE Patients

Fibrinogen is a glycoprotein complex that is enzymatically converted by thrombin to fibrin at the time of tissue injury, causing the blood to clot and stop bleeding.^29^ Laboratory parameters of COVID-19 patients have shown a prothrombic diathesis with significantly high fibrinogen levels in critically ill patients.^38^ Giannis and Ziogas^30^ examined the effect of COVID-19 on the coagulation cascade in-vitro. They reported a high expression of genes for the procoagulant factor fibrinogen (FGB, FGG) in the mononuclear cells infected by the virus. A related analysis by Zou et al^33^ assessed the coagulation function of 303 COVID-19 patients. The results indicated that fibrinogen levels >7.0 g/L in 5.7% of the patients with mild disease as compared to 19.1% with severe disease. However, in the late stages, thrombolysis decreases fibrinogen levels and increases fibrin-degradation products. If coagulation remains unchecked, it can lead to consumptive coagulopathy and bleeding, as demonstrated by Connors and Levy.^1^

## Practical Recommendation and International Guidelines for the Management of VTE in COVID-19 Patients

**Figure 1:**
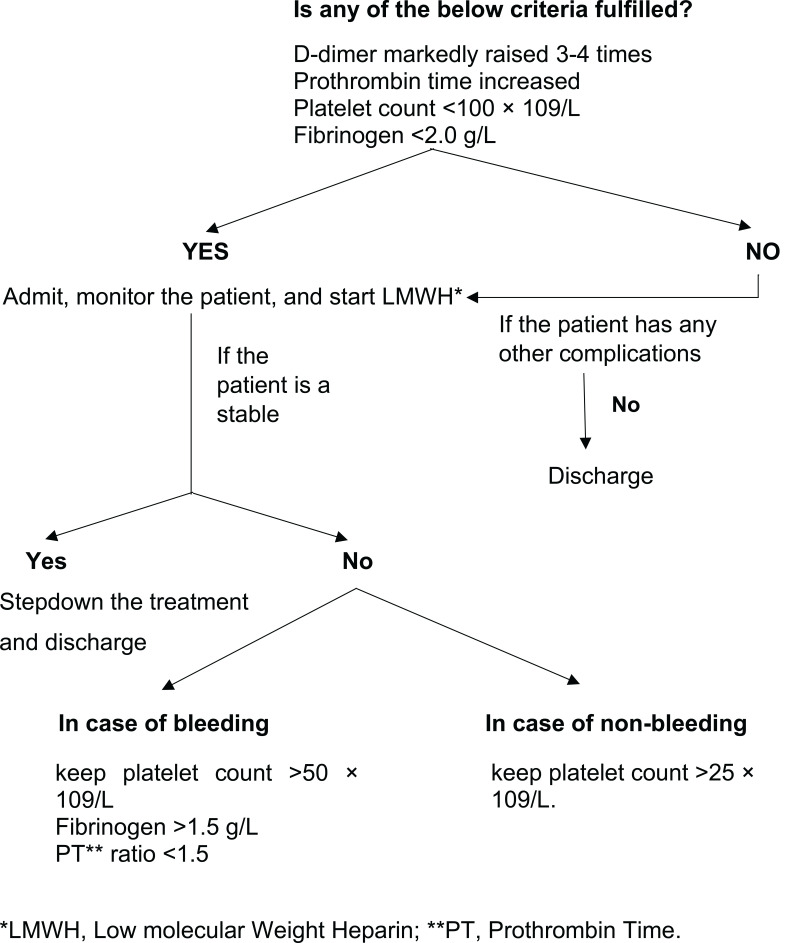
Algorithm for the management of coagulopathy in COVID-19 based on simple laboratory markers.**Note:** Data from Thachil J, Tang N, Gando S, et al.ISTH interim guidance on recognition and management of coagulopathy in COVID-19. *J Thromb Haemost*. 2020;18(5):1023–1026. doi:10.1111/jth.14810.^27^

COVID-19 is a systemic inflammatory infection in which mortality is determined by the degree of inflammation and coagulation. Therefore, prevention, early diagnosis, and prompt treatment of VTE are critical to saving lives. All hospitalized COVID-19 patients must have a routine risk assessment for VTE.^28^ Figure 1 shows the International Society on Thrombosis and Haemostasis (ISTH) algorithm for managing coagulopathy in COVID-19 patients based on laboratory parameters: D-dimer, PT, aPTT, platelets, and fibrinogen.27 Markedly raised D-dimer, prolonged PT, platelet count <100 x 109 and fibrinogen <2.0 g/L call for hospital admission and prophylactic administration (Thromboprophylaxis) of low molecular weight heparin (LMWH) or unfractionated heparin (UFH) in all patients (critical and non-critical) even in the absence of any other comorbidity and absolute contraindications (active bleeding or severe thrombocytopenia). Liver and renal function tests need to be considered and monitored when determining the appropriate dose and type of anticoagulant medications. Additionally, regular monitoring of all the parameters once or twice daily for proper assessment of disease progression.

A study^32^ presented a difference in the incidence of DVT in prophylactic and non-prophylactic subgroups (18% and 35%), respectively. Therefore, it is evident that VTE’s risk remains high in critically ill COVID-19 patients, and thromboprophylaxis reduces mortality due to thrombotic events. Another study detected that heparin decreased mortality risk in patients who have met the sepsis-induced coagulopathy (SIC) criteria ≥4 and patients who have met the sepsis-induced coagulopathy (SIC) criteria <4 by 37.5% and 24%, correspondingly.^39^ Similar results were found in the past investigations of the biology of SARS viruses in which heparin showed a 50% reduction in SARS-CoV2 infectivity.^33^

Anticoagulation with heparin seems to be the mainstay of treatment of critically ill COVID-19 patients with VTE because of its antithrombotic, anti-inflammatory, anti-complement, and direct anti-viral effects. Heparin inhibits neutrophil activation, binds inflammatory cytokines, and reduces endothelial activation hence blocking the cytokine storm that is the primary pathogenic event behind VTE in COVID-19.^34^ Moreover, it has some cardiac benefits and antiviral role as showed in vitro and animal studies, but the clinical benefits are not yet investigated.^39^

## Conclusion

The COVID-19 pandemic has put medical knowledge to the challenge of finding treatment for a pathogen whose behavior is yet to be defined. Evaluation of the results from studies on VTE in critically ill COVID-19 patients has led us to conclude that inflammatory hyper-responsiveness leading to cytokine storm is the primary pathogenic event behind increased risk for VTE and mortality in hospitalized patients. The pathogenic events manifest as deranged laboratory parameters, including fibrinogen, D-dimers, IL-6. Therefore, prophylaxis, early diagnosis, and prompt treatment remain the pillar of the management of VTE in COVID-19 patients and reducing mortality.
